# Disseminated Intravascular Coagulation after Surgery for Facial Injury

**DOI:** 10.1155/2016/6053652

**Published:** 2016-05-30

**Authors:** Hisashi Ozaki, Hirohiko Tachibana, Shigeo Ishikawa, Kazuyuki Yusa, Kenichirou Kitabatake, Mitsuyoshi Iino

**Affiliations:** Department of Dentistry, Oral and Maxillofacial-Plastic and Reconstructive Surgery, Faculty of Medicine, Yamagata University, 2-2-2 Iida-Nishi, Yamagata 990-9585, Japan

## Abstract

A case of disseminated intravascular coagulation (DIC) presenting after surgery for facial trauma associated with multiple facial bone fractures is described. With regard to the oral and maxillofacial region, DIC has been described in the literature following head trauma, infection, and metastatic disease. Until now, only 5 reports have described DIC after surgery for facial injury. DIC secondary to facial injury is thus rare. The patient in this case was young and had no medical history. Preoperative hemorrhage or postoperative septicemia may thus induce DIC.

## 1. Introduction

Disseminated intravascular coagulation (DIC) is a dynamic pathologic process in which thrombin forms within the vascular system [1]. DIC is commonly associated with malignant neoplasm, major trauma, head injury, infection, and obstetric complications [2]. With regard to the oral and maxillofacial region, DIC has been described in the literature following head trauma, infection, and metastatic disease [3]. However, a review of literature shows only a small number of reports describing DIC in relation to oral and maxillofacial surgery [4]. In particular, few reports have mentioned DIC after surgery for facial injury. We report herein a rare case of DIC after surgery for facial trauma associated with multiple facial bone fractures.

## 2. Case Presentation

**Figure 1:**
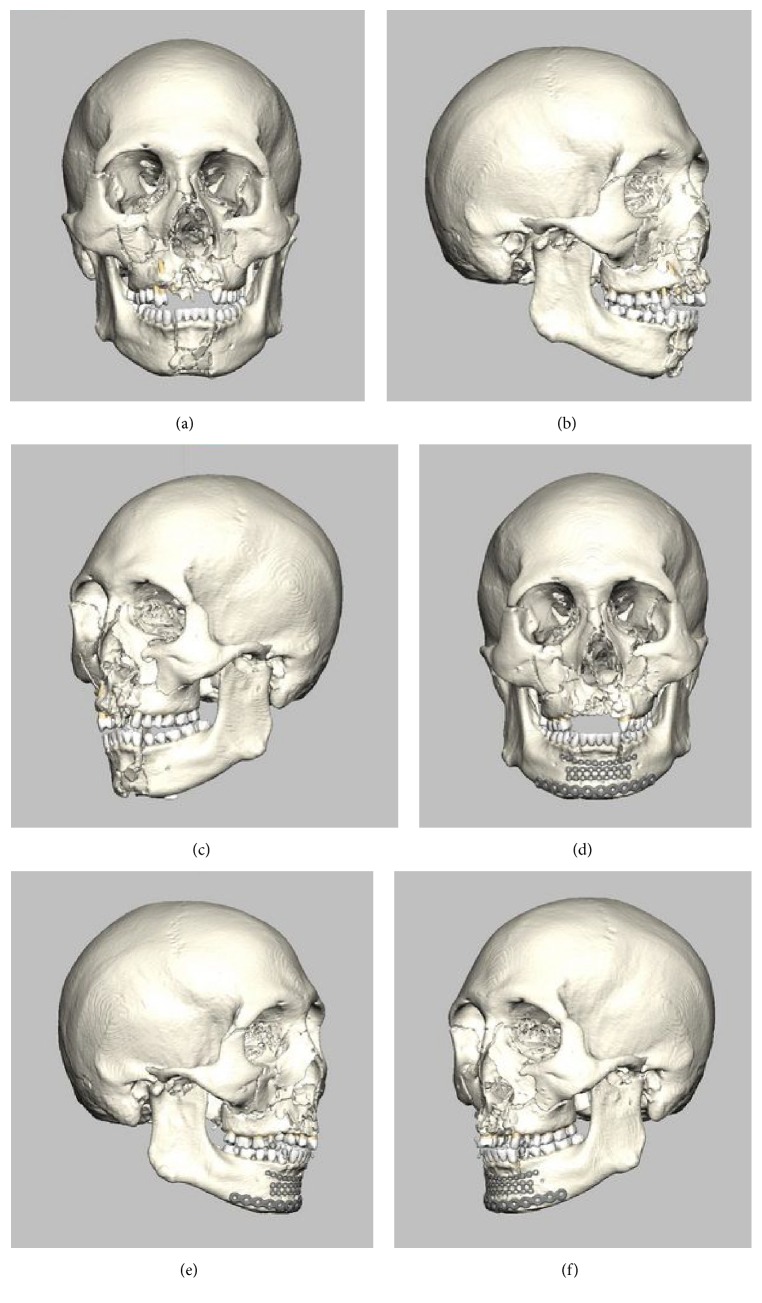
Images from preoperative and postoperative 3D-CT. (a)–(c) showed that bilateral condylar and mandibular fractures, LeFort II-type fracture, and blow-out fracture of orbit are apparent. (d)–(f) showed that mandibular fracture was fixed by titanium plates and LeFort II-type fracture and blow-out fracture of the orbit were fixed by absorbable plates. (a) Preoperative frontal view. (b) Preoperative lateral view of the right. (c) Preoperative lateral view of the left. (d) Postoperative frontal view. (e) Postoperative lateral view of the right. (f) Postoperative lateral view of the left.

A 21-year-old man was brought to the emergency department at Yamagata University Hospital after becoming involved in a traffic accident while riding a motorbike. On arrival, he was fully conscious and complained of facial pain. Clinical examination showed facial swelling, persistent intraoral and nasal hemorrhage, and bloody otorrhea from the left ear. He showed gross malocclusion associated with discontinuity and mobilization of the maxillary and mandibular dentitions. He had no significant medical history and had been healthy before the accident. Computed tomography (CT) and plain radiography revealed right pulmonary contusion, fractures of a second rib and the right radius, and airway narrowing. In the maxillofacial region, bilateral condylar and mandibular fractures, LeFort II-type fracture, and blow-out fracture of the orbit were recognized (Figure 1). Airway control was achieved by awake orotracheal intubation. Hemostasis was performed by suture compression for oral hemorrhage and by gauze tamponade for nasal hemorrhage under local anesthesia, although nasal hemostasis proved extremely difficult to achieve. Respiratory management with a ventilator was conducted under intravenous sedation until general condition was stable. After another 2 days, the tracheal tube was removed, because no airway narrowing or obstruction was evident. Six days after the accident, tracheotomy and repositioning and fixation of the fractured facial bones, including the mandible, maxilla, zygoma, and blow-out fracture of the orbit, were performed under general anesthesia (Figure 1). Surgery lasted 7 h 16 min, with 30 mL of intraoperative bleeding. Intraoperatively, the patient received blood transfusion of 2 units of red blood cell concentrate due to low hemoglobin levels (7.3 g/dL). The postoperative course was uneventful. On postoperative day 5, however, fever over 39.0°C and shivering were noted. Laboratory examination showed increases in the white blood cell count to 14,060/*μ*L and C-reactive protein (CRP) to 12.1 mg/dL and a decrease in platelets to 131 × 10^3^/*μ*L (down from 238 × 10^3^/*μ*L at the time of the accident). CT revealed no abnormalities other than those at the surgical sites. The next day, the patient showed preshock status with a significant decrease in blood pressure, fever over 39.0°C, and transient loss of consciousness. Emergency laboratory tests showed increases in CRP to 23.9 mg/dL, TBIL to 2.1 mg/dL, creatinine to 0.97 mg/dL, lactate to 4.07 mmol/L, prothrombin time to 21.7 s, FDP (fibrin degradation product) to 10.8 *μ*g/mL, and PT-INR (prothrombin time-international normalized ratio) to 1.86, along with a decrease in platelets to 46 × 10^3^/*μ*L (Table 1). The bacteria was not detected in several tests of blood culture. In the evaluation of respiratory system, PaO2 was 68.2 mmHg, PaCO2 was 28.2 mmHg in blood gas analysis, and PaO2/FiO2 was 341. In circulatory dynamics, noradrenaline was administered sustainably by 0.04*ɤ* for keeping of blood pressure. In the evaluation of central nervous system, Glasgow Coma Scale was E4V5M6 and a total of 15. Therefore, SOFA (sequential organ failure assessment) score was 9. From the above results, septic shock was diagnosed and the patient was sent to the intensive care unit (ICU). According to the scoring algorithm criteria established by the Japanese Association for Acute Medicine (JAAM) for DIC [21], the patient scored 5. DIC was therefore diagnosed and treatment was initiated. To treat severe infection, cefozopran hydrochloride and freeze-dried polyethylene glycol-treated human normal immunoglobulin were applied by intravenous injection empirically. A total of 38,840 units of thrombomodulin *α* was administered for the treatment of DIC. After another 7 days, laboratory tests showed a return to nearly normal state, with CRP of 4.09 mg/dL; TBIL of 0.6 mg/dL, Crea of 0.67 mg/dL, WBC of 12,160/*μ*L; platelet count of 175 × 10^3^/*μ*L; prothrombin time of 13.7 s; FDP of 5.3 *μ*g/mL; and PT-INR of 1.17 (Table 1). The patient was then moved to a general ward and the subsequent course was uneventful.

## 3. Discussion

In the oral and maxillofacial region, several reports have described DIC in patients with oral cancer [17], infection [11], injury [3, 13, 16, 17], orthognathic surgery [12], and tooth extraction [5–10, 14, 15, 18, 19]. To the best of our knowledge, however, the English literature contains only 19 reports of DIC in relation to oral and maxillofacial surgery except for oral cancer (Table 2). This means that DIC associated with oral and maxillofacial surgery is uncommon. Of these 19 cases, the most frequent surgery associated with DIC was tooth extraction, in 10 cases. On the other hand, including our case, only 5 cases involved facial injury related to DIC. Table 2 also shows the diseases underlying DIC, with prostatic adenocarcinoma and aortic aneurysm in 4 cases each, and septicemia in 3 cases. Among the 5 cases of facial injury, each patient showed prostatic adenocarcinoma, abruption, septicemia, or no underlying disease. In the remaining cases, Morimoto et al. [17] did not discuss the causative underlying disease. The present patient had no medical history before the traffic accident and preoperative hemostatic function tests yielded normal results. Two reasons could explain DIC in the present case. One is septicemia associated with severe postoperative systemic inflammatory response syndrome (SIRS). Postoperative stress seemed to not only induce SIRS but also increase its severity. Under severe SIRS, DIC may have developed from hypercoagulability because of abnormally high activity of a chemical mediator [22]. The other is persistent intraoral and nasal hemorrhage after the accident. Laboratory testing showed prominent decreases in hemoglobin (14.7 g/dL to 7.3 g/dL) and platelets (23.8 × 10^4^/*μ*L to 9.8 × 10^4^/*μ*L) between the first visit and the surgery. DIC was possibly triggered in the patient from the large volume of blood loss, consumption of platelets and coagulation factors by acute massive hemorrhage, and a high volume of blood transfusion within a short period [22, 23]. Under such general conditions, the patient had already been in a chronic state of DIC before surgery, and this served to aggravate the symptoms.

The occurrence of DIC is difficult to predict in cases involving young patients with no underlying disease, as in the present case. Over the last 20 years, no fatal cases of DIC have been reported in association with oral and maxillofacial surgery (Table 2). Therefore, in cases with facial injury, symptoms must be detected and progression of DIC prevented by careful perioperative management.

## Figures and Tables

**Table 1:** Laboratory data.

Examination	First visit	Preoperative	POD 5	POD 6 (preshock)	POD 13
WBC (×10^3^/*μ*L)	17.71	10.09	14.06	10.17	12.16
RBC (×10^6^/*μ*L)	4.69	2.33	2.91	2.7	2.55
Hemoglobin (g/dL)	14.7	7.3	9.0	8.2	7.7
Hematocrit (%)	44	22.3	26.4	25	24.1
Platelets (×10^4^/*μ*L)	23.8	9.8	13.1	4.6	17.5
CRP (mg/dL)	<0.10	3.82	12.10	23.9	4.09
TBIL (mg/dL)	0.8	0.4	—	2.1	0.6

Crea (mg/dL)	0.94	0.64	0.76	0.97	0.67

BUN (mg/dL)	16	14	20	30	13

Lactate (mmol/L)	4.07	—	—	4.07	—

Glucose (mg/dL)	169	—	—	118	—

PT (s)	—	11	—	21.7	13.7
PT (%)	—	110	—	43	79
PT-INR	—	0.94	—	1.86	1.17
aPTT (s)	—	27.8	—	35	30.4
Fbg (mg/dL)	—	—	—	664	624
FDP (*μ*g/mL)	—	—	—	10.8	5.3
D-dimer (*μ*g/mL)	—	—	—	6.4	5.07

POD: postoperative day.

**Table 2:** DIC relevant to oral and maxillofacial surgery.

Number	Author	Year	Age	Sex	Surgery	Underlying disease	Progress
1	Falace and Kelly [5]	1976	85	M	Extraction of 6 anterior teeth	Occult prostatic adenocarcinoma	Alive
2	Rawson et al. [6, 7]	1976	45	F	Extraction of a third molar and submandibular abscess	Septicemia	Dead
3	Samman [3]	1984	28	F	Forehead, lip, and tongue laceration from traffic accident	Abruption (38-week pregnancy)	Alive
4	McKechnie [8]	1989	59	M	Extraction of maxillary first molar	Occult prostatic adenocarcinoma	Dead
5	Chishiro [9]	1989	86	M	Extraction of maxillary molar	Known abdominal aortic aneurysm	Dead
6	Marshall et al. [10]	1993	24	F	Extraction of 3 third molars	Unknown	Dead
7	Currie and Ho [11]	1993	31	M	Acute dentoalveolar abscess	Septicemia	Dead
8	Christiansen and Soudah [12]	1993	19	M	LeFort I osteotomy	None	Alive
9	McLoughlin et al. [13]	1994	62	M	Lower lip laceration	Occult prostatic adenocarcinoma, multiple bone metastases	Alive
10	Herold and Falworth [14]	1994	73	M	Extraction of upper canine	Occult abdominal aortic aneurysm	Alive
11	Mehra et al. [4]	1997	65	M	Scaling, parotitis	Unknown	Alive
12	Sawaki et al. [15]	1999	62	M	Extraction of maxillary premolar and molar	Occult prostatic adenocarcinoma, multiple bone metastases	Alive
13	Cbo et al. [16]	2001	28	F	Fracture of mandible	None	Alive
14	Morimoto et al. [17]	2001	37	F	Infection after tooth extraction	Unknown	Alive
15	Morimoto et al. [17]	2001	25	M	Multiple injuries of the mandible	None	Alive
16	Ita et al. [18]	2001	22	F	Extraction of mandibular third molar	KTW syndrome	Alive
17	Peters et al. [19]	2005	82	M	Extraction of anterior tooth	Aortic aneurysm	Alive
18	Imai et al. [20]	2010	88	M	Spontaneous hemorrhage	Aortic aneurysm	Alive

19	Ozaki et al.	2016	21	M	Multiple facial injury from traffic accident	Septicemia	Alive
